# Development of real-time PCR and loop-mediated isothermal amplification (LAMP) assays for the differential detection of digital dermatitis associated treponemes

**DOI:** 10.1371/journal.pone.0178349

**Published:** 2017-05-25

**Authors:** Kelly Anklam, Megan Kulow, Wataru Yamazaki, Dörte Döpfer

**Affiliations:** 1 Department of Medical Science, School of Veterinary Medicine, University of Wisconsin, Madison, Wisconsin, United States of America; 2 Department of Veterinary Science, Faculty of Agriculture, University of Miyazaki, Miyazaki, Japan; University of Helsinki, FINLAND

## Abstract

Bovine digital dermatitis (DD) is a severe infectious cause of lameness in cattle worldwide, with important economic and welfare consequences. There are three treponeme phylogroups (*T*. *pedis*, *T*. *phagedenis*, and *T*. *medium*) that are implicated in playing an important causative role in DD. This study was conducted to develop real-time PCR and loop-mediated isothermal amplification (LAMP) assays for the detection and differentiation of the three treponeme phylogroups associated with DD. The real-time PCR treponeme phylogroup assays targeted the 16S-23S rDNA intergenic space (ITS) for *T*. *pedis* and *T*. *phagedenis*, and the flagellin gene (*flaB2*) for *T*. *medium*. The 3 treponeme phylogroup LAMP assays targeted the flagellin gene (*flaB2*) and the 16S rRNA was targeted for the *Treponeme ssp*. LAMP assay. The real-time PCR and LAMP assays correctly detected the target sequence of all control strains examined, and no cross-reactions were observed, representing 100% specificity. The limit of detection for each of the three treponeme phylogroup real-time PCR and LAMP assays was ≤ 70 fg/μl. The detection limit for the *Treponema spp*. LAMP assay ranged from 7–690 fg/μl depending on phylogroup. Treponemes were isolated from 40 DD lesion biopsies using an immunomagnetic separation culture method. The treponeme isolation samples were then subjected to the real-time PCR and LAMP assays for analysis. The treponeme phylogroup real-time PCR and LAMP assay results had 100% agreement, matching on all isolation samples. These results indicate that the developed assays are a sensitive and specific test for the detection and differentiation of the three main treponeme phylogroups implicated in DD.

## Introduction

Bovine digital dermatitis (DD) is a worldwide disease causing severe lameness in cattle in all production systems [1,2]. The disease is characterized by circumscribed ulceroproliferative lesions typically located on the plantar aspect of the hoof [3,4]. The consequences of DD are decreased animal welfare and economic loss due to reduced milk production, decreased reproductive performance and premature culling [5–7].

The cause of DD is multifactorial with an essential spirochetal bacterial component [8,9]. The interaction of the causative factors, including the host, spirochetes and an unhygienic environment result in DD. The spirochetal bacterial component of DD is from the genus *Treponema* and is considered to be polytreponemal in etiology [10,11]. The most highly associated phylogroups of DD are, *Treponema phagedenis*-like, *Treponema medium/vincentii*-like, and *Treponema denticola/putidum*-like, and with the latter also recognized as a new species, *Treponema pedis* [12–14]. Recent sequence analysis studies suggest the removal of the “-like” suffix for the previously mentioned phylogroups [15,16]. Therefore we will use the following nomenclature in this study for the three phylogroups associated with DD: *T*. *pedis*, *T*. *phagedenis*, and *T*. *medium*. According to Moter et al. (1998) [17] the presence of certain treponema phylogroups may correlate with the invasiveness of the disease. Therefore, detecting and differentiating treponema phylogroups in lesion biopsies would be useful for studies of epidemiology and pathogenesis of DD.

There are several conventional PCR based methods used to differentiate the treponeme phylogroups [12,18]. However to date there are no real-time PCR or loop-mediated isothermal amplification (LAMP) assays to differentiate the *Treponema* phylogroups. Compared to conventional PCR, real-time PCR and LAMP assays are more specific and sensitive and are less labor intensive. Real-time PCR can detect the amplification of products, as the products are synthesized. With the development of technology, PCR has become a very popular technique, especially for the detection and identification of bacteria. The real-time PCR uses a florescent dye system and thermocycler equipped with fluorescent- detection capability. The LAMP assay is a nucleotide acid amplification method that features high sensitivity, specificity, and rapidity under isothermal conditions [19]. LAMP requires a set of four primers to react with six distinct regions in the target. The reaction can be accelerated by adding two loop primers. The LAMP assay utilizes a water bath or heat block for amplification thus avoiding the dependency on a thermocycler or electrophoresis equipment. The interpretation of the reaction can be observed by the naked eye with visual fluorescence. In this study we propose to develop and evaluate real-time PCR and LAMP assays capable of detecting and differentiating treponemes associated with DD.

Treponemes are notoriously difficult to cultivate and isolate [20]. Isolation attempts fail typically due to high level of contamination, as opposed to the absence of treponemes. PCR based methods circumvent the difficulties associated with cultivation of the fastidious treponemes, however PCR detection does not prove viability. The use of improved treponeme isolation methods and the development of detection methods are warranted. Immunomagnetic separation (IMS) has been used successfully to isolate spirochetes from cattle with DD and sheep with ovine foot rot [21,22]. Developing an improved treponeme isolation method would help increase our understanding of the pathogenesis of DD. In this study we will use a similar IMS method to improve treponeme isolation from DD lesions for future pathogenesis and transmission research. The objective of this study was to develop phylogroup specific treponeme real-time PCR and LAMP assays and evaluate the real-time PCR and LAMP assays based on specificity and sensitivity.

## Methods and materials

### Bacterial strains

Thirty-four treponeme strains isolated from DD lesions and previously characterized and twenty negative bacterial control strains were used to evaluate the real-time PCR and LAMP assays. Treponeme strains were cultured anaerobically (85% N_2_, 10% H_2_, and 5% CO_2_) at 37°C in oral treponeme enrichment broth (OTEB, Anaerobe Systems, Morgan Hill, CA) supplemented with 10% fetal bovine serum (FBS, GE Healthcare Life Sciences, Marlborough, MA) for 7–10 days. The negative controls strains were cultured in Luria-Bertani (LB) broth (Becton Dickinson, Sparks, MD) and incubated at 37°C for 18–20 h or cultured in recommended bacterial strain specific media and conditions.

### DNA extraction

DNA was extracted from treponeme cultures and other bacterial cultures using the QIAmp DNA mini-prep kit (Qiagen, Valencia, CA) according to the manufacturer’s instructions or by heat lysis. The heat lysis protocol for DNA extraction was performed as follows: one ml of culture was centrifuged for 5 min at 9,300 x g and the supernatant was discarded. The remaining pellet or a colony was resuspended in 50 μl of nuclease free water and vortexed vigorously. The suspension was incubated on a dry heat block for 20 minutes at 100°C, and then centrifuged for 10 min at 20,000 x g. The resulting supernatant (DNA template) was transferred to a labeled microcentrifuge tube and stored at -20°C.

### Real-time PCR

The primer:probe sets were designed to target the 16S-23S rDNA intergenic space (ITS) 16S-tRNA^Ile^ for *T*. *pedis* and *T*. *phagadenis*, and the flagellin gene (*flaB2*) for *T*. *medium* Table 1. The real-time PCR assays were performed in 25 μl reactions containing: 2.5 μl of extracted DNA template, 12.5 μl of 2X QuantiTect Multiplex PCR with ROX Master-mix (Qiagen, Valencia, CA), 1 μl of each primer:probe (Eurofins MWG Operon, Louisville, KY) mix containing a final primer concentration of 0.4 μM and final probe concentration of 0.1 μM and sterile PCR grade water (Promega, Madison, WI). PCR reactions were performed on a Stratagene MX3005P^TM^ qPCR system (Agilent Technologies, LaJolla, CA). The PCR protocol consisted of an initial denaturation at 95°C for 10 minutes followed by 40 cycles of 95°C for 30 seconds and 60°C for 1 minute and the real-time fluorescence data acquisition occurred at the end of each annealing/extension phase. All PCR assays use the same PCR cycling conditions allowing parallel testing of the 3 PCR assays.

**Table 1:** Primers and fluorescence-labeled oligonuclueotide probes for the three real-time PCR assays.

Target Gene	Primer/Probe Specificity	Primer/Probe Sequence (5'-3')	Amplicon (bp)	Position	Accession Number
16S-tRNA^Ile^ region	*T*. *pedis*	TTGAAGTACACAAGACGCTC		109–128	AF179255
		CCCCTTCCTTATCAGAGAA	118	226–208	
		FAM GTGCTCTAACCAACTGAGCTACAGGC BHQ1		207–182	
16S-tRNA^Ile^ region	*T*. *phagedenis*	GTCTATACTCTTAAAACGATGCGC		87–110	AF179261
		CCCCTTCCTTATCAGAGAA	104	190–172	
		FAM GTGCTCTAACCAACTGAGCTACAGGC BHQ1		171–146	
*flaB2*	*T*. *medium*	CGATACGCCTGAAACAGC		369–386	EF061271
		TACCGACAACACTCATTTCG	122	490–471	
		FAM TGAAGCCATCAAGAAGATCAACAAGCAGCG BHQ1		411–440	

### LAMP primer set design

Primer sequences are listed in Table 2. The primers were designed according to the instructions (http://loopamp.eiken.co.jp/e/lamp/primer.html), in terms of the distance between primers, Tm value for primer regions, GC contents, and the stability of primer end and secondary structure of primers. Primer sets were designed to target the flagellin gene (*flaB2*) for *T*. *pedis*, *T*. *phagedenis*, and *T*. *medium*. The *Treponema* species primer set targets the 16S rRNA and was designed to encompass the predominant DD associated treponemes, *T*. *pedis*, *T*. *phagedenis*, and *T*. *medium*.

**Table 2:** Primers for the four LAMP assays.

Target Gene	Primer Specificity	LAMP Primer	Primer Sequence (5'-3')
16S rRNA	*Treponema spp*.^a^	F3	CCCTTAAGATGGGGATAGCT
		B3	CCATTGCGGAATATTCTTAGCT
		FIP	CCGTTACCTCACCAACAAGCATAA-TAAAGCCGTATAAGGAAAGGAG
		BIP	CCTGAGAGGGTGGACGGACA-TGCCTCCCGTAGGAGTTTG
		LF	ACGCGGGCTCATCCTCAAG
		LB	CATTGGGACTGAGATACGGCC
*flaB2*	*T*. *pedis*	F3	CAAACATGGACCAAAGAATGC
		B3	CGTAAGTTCCATTCTGTTCTG
		FIP	TCGGCAGTTTCGATTGTCATAA-TCGGAACAATGTCGGCTG
		BIP	TTCCGCCAATATGAGCATCG-TCCGAGGTCCGCTCTTT
		LF	CTTTTCCGAACCGATTTCGC
		LB	GAACGATTGATGAAGGCTTAAAG
*flaB2*	*T*. *phagedenis*^b^	F3	CACTGTTACCGCTTCTATGTG
		B3	CGCCGATAACAGTGTACTTG
		FIP	GATTCATCCCCAACATCGC-ACATGGACCAGAGAACACG
		BIP	CGCAATCGGTACTCTTGATG-TGTTCTGGTATGCACCGAG
		LF	GCAGTCATTGTTCCGATGTAT
*flaB2*	*T*. *medium*	F3	CGTGCGTATGTCGGTACA
		B3	GCAACATTGATACCGACAACA
		FIP	GCTGTTTCAGGCGTATCG-CGCTAAAGCACTTGGTGTTC
		BIP	TGAAGCCATCAAGAAGATCAAC-CTCATTTCGAGTCTGTTCTG
		LF	GATTCGTCACCAATGTCGC
		LB	GCTGATCTCGGTGCATAC

^**a**^The *Treponema* species primer was designed to encompass *T*. *pedis*, *T*. *phagedenis*, and *T*. *medium*.

^b^There is no LB primer for the *T*. *phagedenis* due to unfavorable sequence in that location. The loop primers (LB and LF) are not necessary for the LAMP assay to perform however the loop primers increase efficiency.

### LAMP assays

LAMP assays were conducted as previously described [19] with some modifications. Briefly, each reaction was performed in a total of 25 μl mixture containing 1.6 μM (each) of the primers FIP and BIP, 0.2 μM (each) of the primers F3 and B3, 0.8 μM (each) of the primers LF and LB (Eurofins MWG Operon, Louisville, KY), 1.2 mM deoxynucleotide triphosphates (Promega, Madison, WI), 6mM MgSO_4_ (Sigma-Aldrich, St. Louis, MO), 1 M betaine (MP Biomedicals, Solon, OH), 1x thermopol buffer (New England BioLabs, Ipseich, MA), 8 U *Bst* DNA polymerase (New England BioLabs, Ipseich, MA), sterile PCR grade water (Promega, Madison, WI), and 2 μl of DNA template. The reaction was amplified for 45 minutes at 65°C and was terminated by heating at 80°C for 2 minutes. After the amplification, 1 μl SYBR Green I (Lonza, Allendale, NJ) was added to each LAMP reaction tube to observe the color change. Since fluorescent dye SYBR Green I binds to double-stranded DNA and produces a yellow fluorescence which can be observed by the naked eye under natural light or under UV lamp. The observation of the yellow/fluorescence color change indicates a positive reaction.

### Specificity of the treponeme phylogroup real-time assays

The specificity of the three treponeme phylogroup assays was assessed by testing a panel of 60 positive and negative control strains Table 3. A cycle threshold (Ct) of <38 was the cutoff for positive samples, corresponding to the reliable limit of detection of the assays based on recommendations by the World Organization of Animal Health (Paris, France).

**Table 3:** Bacterial strains used for the development of the real-time PCR and LAMP assays.

Bacterial strain	No. of strains tested
*T*. *pedis*^a^^,^^b^	6
*T*. *phagedenis*^a^^,^^b^	18
*T*. *medium*^a^	10
*Bacillus cereus*^d^	1
*Bacillus fragilis*^d^	1
*Borrelia burgdorferi*^c^	1
*Brachspira hyodysenteriae*^c^	1
*Fusobacterium necrophorum*^c^	1
*Fusobacterium nucleatum*^c^	1
*Porphyromonas levii*^c^	1
*Dichelobacter nodosus*^c^	1
*Prevotella denticola*^c^	1
*Streptococcus dysgalactiae*^d^	1
*Citrobacter freundii*^e^	1
*Enterobacter taylorae*^e^	1
*Entrobacter aerogenes*^d^	1
*Enterococcus feacalis*^c^	1
*Escherichia coli*^c^^,^^e^	2
*Klebsiella ozanae*^e^	1
*Leptospira interrogans*^d^	3
*Listeria moncytogenes*^d^	1
*Pseudomonas aeruginosa*^c^	1
*Salmonella enteriditis*^d^	1
*Staphylococcus aureus*^c^	1
*Shigella dysenteriae*^c^	1
*Yersinia enterocolitica*^c^	1

^a^University of Wisconsin-Madison—Döpfer, Madison, WI

^b^University of California-Davis, Davis, CA.

^c^American Type Culture Collection, Manassas, VA

^d^Veterinary Medicine Teaching Hospital, Madison, WI

^e^Food Research Insitiute–Kasper, Madison, WI

### Limit of detection of the real-time PCR assays

Template DNA extracted from treponeme cultures of each of three phylogroup strains was 10-fold serially diluted and quantified by using the Quant-iT^™^ dsDNA high-sensitivity assay kit (Molecular Probes, Eugene, OR) with a fluorescence microplate reader (Stratagene MX3005P^™^ qPCR system, Agilent Technologies, LaJolla, CA). The Ct values were determined for each dilution in duplicate. Standard curves were constructed by plotting the Ct values obtained from amplification of each phylotype target sequence against DNA concentration (log femtogram (fg) per microliter) of each dilution. The regression lines, slopes of the regression lines, and correlation coefficients (R^2^) were derived using Prism 2004 software (Graphpad Software, Inc., San Diego, CA). PCR efficiencies were calculated with the following formula: *E* = 10^(-1/slope)^. The limit of detection was determined to be the last duplicate positive reaction (Ct <38) detected in the series.

### Specificity and limit of detection of the LAMP assays

For the four LAMP assays specificity was assessed by testing the same panel of 60 positive and negative control strains that were previously used for the real-time PCR assays Table 3. The LAMP assays limit of detection was determined by template DNA extracted from treponeme cultures of each of three phylogroup strains was 10-fold serially diluted and quantified by using the Quant-iT^™^ dsDNA high-sensitivity assay kit (Molecular Probes, Eugene, OR) with a fluorescence microplate reader (Stratagene MX3005P^™^ qPCR system, Agilent Technologies, LaJolla, CA). The detection limit was examined by analyzing the products yield from the 10-fold serial dilutions in duplicate. The positive reactions were visualized as a fluorescence color change under an UV lamp. The limit of detection was determined to be the last duplicate positive reaction observed in the series.

### Anti-treponema antibody preparation for immunomagnetic separation of treponemes

The cellular antigens extracted from 1-9185MED *T*. *pedis* and 2–1498 *T*. *phagedenis* were supplied to a commercial custom antisera service (Panigen, Blanchardville, WI) for the generation of rabbit antisera. The antisera were tested for reactivity by ELISA against the antigens of *T*. *pedis T*. *phagedenis*, *and T*. *medium* and were shown to cross-react with the three phylotypes. Antibody purification was performed on the antisera by using Magne^™^ Protein A beads (Promega, Madison, WI) according to the manufacturer’s instructions. The recovered antibody was quantified by using a protein assay kit (Thermo Scientific, Rockford, IL) in accordance with the manufacturer’s instructions and stored at -20°C until usage. The purified antibody was covalently coupled to Dynabeads^®^ M-270 epoxy in accordance with the manufacturer’s instructions. Briefly, 300 μg of purified 1-9185MED *T*. *pedis* or 2–1498 *T*. *phagedenis* anti-treponeme antibody was added to 5 mg of beads and two coupling buffers and incubated overnight at 37°C with slow tilt rotation. After incubation the antibody coupled beads were captured by the use of a magnetic stand (DynaMag^™^, Dynal, Oslo, Norway) and washed four times with wash buffers. The antibody coupled beads were resuspended in 500 μl of wash buffer and a stored at 2°C—8°C until used.

### DD biopsy survey

For this study, a 5-point scale according to Dopfer et al. (1997) [23] and Berry et al. (2012) [24] was used to classify the DD lesions of a total of 40 beef cattle of multiple breeds from a commercial feedlot. Lesions were classified M1 and M4.1 if a DD lesion <20 mm in diameter was observed surrounded by healthy skin or embedded in a circumscribed dyskeratotic or proliferative skin alteration, respectively, M2 if an active lesion was found with diameter ≥20 mm, and M4 if only a circumscribed dyskeratotic or proliferative skin alteration was identified. Based upon the number of active M2 lesions identified during the study, all animals were further classified as type I (no M2 lesions identified), type II (only one M2 event) or type III (multiple M2 lesions observed). DD lesions were biopsied using a 3 mm punch biopsy under local anesthesia or immediately after being euthanized at slaughter by either exsanguination or captive bolt at the Aurora Packing slaughter house in Illinois.

This study was carried out in strict accordance with the recommendations in the Guide for the Care and Use of Agricultural Animals in Agricultural Research and Teaching. The protocol was approved by the Institutional Animal Care and Use Committee Research of the Animal Resource Center at the University of Wisconsin (Protocol Number: V01525-0-02-14). All biopsies were performed under local anesthesia by intravenous regional analgesia, and all efforts were made to minimize suffering.

Tissue biopsy samples were transferred into transport medium and placed on ice for subsequent Treponeme culture. The transport medium consisted of OTEB and contained the antibiotics rifampicin (5 μg/ml, Sigma-Aldrich, St. Louis, MO) and enrofloxacin (5 μg/ml, Sigma-Aldrich, St. Louis, MO). Biopsy samples were minced with a scalpel blade and transferred into a tube containing 3 ml of OTEB and 10% fetal bovine serum (FBS) and the antibiotics rifampicin (5 μg/ml) and enrofloxacin (5 μg/ml). Samples were then incubated for 1 hour at room temperature. All enriched samples were inspected for spirochetes by dark-field microscopy at 20-40X magnification. A 1 ml sample of the enriched biopsy media was transferred in to the anaerobic chamber (85% N_2_, 10% H_2_, and 5% CO_2_, 37°C). The 1 ml enriched biopsy media samples were processed through IMS involving the use of 10 μl of 1-9185MED anti-treponeme antibody coupled beads and 10 μl of 2–1498 anti-treponeme antibody coupled beads per reaction. After an incubation of 10 minutes at room temperature with gentle continuous agitation to prevent the beads from settling, the treponeme-magnetic bead complex was separated from the suspension, washed three times with PBS containing 0.05% Tween-20 (PBST, Amresco, Solon, OH). The treponeme-magnetic bead complex was resuspended in 100 μl PBST and transferred to a tube containing 2 ml of OTEB and 10% FBS and the antibiotics rifampicin (5 μg/ml) and enrofloxacin (5 μg/ml) and incubated for 72 hours at 37°C in the anaerobic chamber. After incubation transfer 500 μl of the enriched treponeme captured culture to a tube containing 3 ml of OTEB and 10% FBS and incubate at 37°C in the anaerobic chamber for ~3–10 days.

The culture was checked for growth and purity by dark-field microscopy and subcultured on fastidious anaerobe agar (FAA, Neogen, Lansing, MI) and subsequently inoculated into 3 ml of OTEB and 10% FBS. DNA was then extracted from the treponeme cultures and the isolated organisms identified using specific treponeme phylogroup PCR and LAMP assays. The total time required for this culturing process varies depending on the phylotype growth pattern, pure isolates may be obtain in 4–6 weeks.

## Results

### Specificity of treponeme phylogroup real-time PCR assays

All of the primers and fluorescence-labeled oligonuclueotide probes were specific to the intended target sequence. The assays correctly detected the phylogroup target sequence of all control strains examined, and no cross-reactions were observed with negative controls, matching 60/60 representing 100% specificity S1 Table. In general, Ct values for positive strains were <20, and no amplification signals were observed for the negative control strains.

### Limit of detection of the treponeme phylogroup real-time PCR assays

To determine the detection limits and quantitative ability of the real-time assays, standard curves were constructed S1 Fig. The standard curves revealed a linear relationship, with slopes of -3.255 to -3.396 and R^2^ = ≥ 0.995, resulting in PCR amplification efficiencies of 1.92 to 2.03. The limit of detection for each of the three Treponeme phylogroup PCR assays was ≤ 70 fg/μl Table 4.

**Table 4:** Detection limits and results of the regression analysis of the real-time PCR assays.

Real-time PCR Target	Detection Limit (fg/μl)	R^2^	Efficiency^a^
*T*. *pedis*	69	0.995	1.99
*T*. *phagedenis*	70	0.997	2.03
*T*. *medium*	59	0.998	1.92

^a^E = 10^(-1/Slope)^, 2.00 = 100% Efficiency

### LAMP assays specificity and limit of detection

Among the panel of 60 control strains used to determine the specificity of the 4 LAMP assays (*Treponema spp*., *T*. *pedis*, *T*. *phagedenis*, and *T*. *medium*), false positives and false negative results were not observed; i.e., the LAMP assays correctly identified 60/60 (100%) of the control strains S1 Table. To estimate the detection limits of the LAMP assays with the primer sets developed in this study, serial DNA dilutions were tested for each of the three phylogroups. The limit of detection for the *T*. *pedis*, *T*. *phagedenis*, and *T*. *medium* phylogroup LAMP assays was 69 fg/μl, 70 fg/μl, and 59 fg/μl, respectively. The detection limit for the *Treponema spp*. LAMP assay ranged from 7–690 fg/μl depending on phylogroup, with *T*. *pedis*, *T*. *phagedenis*, and *T*. *medium* resulting in the following detection limits 690 fg/μl, 7 fg/μl, and 590 fg/μl, respectively.

### DD biopsy survey

For the purpose of demonstrating the applicability of the developed real-time PCR and LAMP assays, treponemes were isolated from 40 DD lesion biopsies that were subjected to IMS. All DD lesion samples were observed to contain treponeme/spirochete organisms microscopically before proceeding to the IMS method. The treponeme phylogroup real-time PCR and LAMP assay results had 100% agreement, matching of 40/40 samples. From the 40 DD lesion IMS cultures all were positive for the *Treponeme spp*. LAMP assay and *T*. *pedis*, *T*. *phagedenis*, and *T*. *medium* phylogroup PCR/LAMP assays were positive for 35%, 85%, and 30% of the samples, respectively Table 5. Multiple phylogroups were detected in 48% of the IMS cultures. Two DD lesion treponeme isolates were negative for the 3 phylogroup PCR/LAMP assays, the 16S rRNA gene was sequenced of each of these isolates and both closely aligned with *Treponema putidum*. The 40 biopsied DD lesions were classified using a 5 point scoring system, 6 of the lesions were classified as M4, 5 of the lesions were classified as M4.1 and 29 of the lesions were classified as M2. The steers of which the lesions were biopsied were also typed based on the number of M2 lesions identified during the study of the 40 steers 28 were Type 2 and 12 were Type 3.

**Table 5:** DD lesion biopsy survey results.

Sample ID	DD Lesion	Chronicity	Steer Type	*Trep*. *spp*. LAMP	*T*. *pedis*	*T*. *phagedenis*	*T*. *medium*
1	M2	None	2	+	-	-	-
2	M2	None	2	+	-	+	-
3	M2	Proliferative	2	+	+	-	+
4	M2	Hyperkeratotic	2	+	+	+	-
5	M2	Proliferative	2	+	+	+	-
6	M2	Proliferative	2	+	-	+	+
7	M2	Proliferative	2	+	-	+	+
8	M2	Proliferative	2	+	-	+	-
9	M2	Proliferative	2	+	-	+	-
10	M4.1	Proliferative	2	+	-	+	+
11	M2	Hyperkeratotic	2	+	+	-	+
12	M2	Proliferative	2	+	+	-	+
13	M2	Hyperkeratotic	2	+	+	+	-
14	M4.1	Proliferative	2	+	+	+	-
15	M2	Proliferative	2	+	+	+	-
16	M4.1	Proliferative	2	+	-	+	-
17	M4.1	Proliferative	2	+	-	+	-
18	M4	Hyperkeratotic	2	+	-	+	-
19	M4	Hyperkeratotic	2	+	-	+	-
20	M2	None	2	+	-	+	-
21	M2	Proliferative	2	+	-	+	-
22	M4.1	Proliferative	2	+	-	+	-
23	M2	Proliferative	2	+	-	+	-
24	M2	Proliferative	2	+	-	+	-
25	M2	Proliferative	2	+	-	+	-
26	M4	Hyperkeratotic	2	+	+	+	+
27	M2	Proliferative	2	+	+	+	+
28	M2	Proliferative	2	+	-	-	-
29	M4	Hyperkeratotic	3	+	-	+	+
30	M2	Proliferative	3	+	+	+	+
31	M2	Proliferative	3	+	+	-	+
32	M2	Proliferative	3	+	+	+	-
33	M4	Proliferative	3	+	+	-	-
34	M4.1	Hyperkeratotic	3	+	-	+	+
35	M2	Hyperkeratotic	3	+	-	+	-
36	M2	None	3	+	-	+	-
37	M2	Proliferative	3	+	-	+	-
38	M2	Proliferative	3	+	-	+	-
39	M2	Proliferative	3	+	-	+	-
40	M2	Proliferative	3	+	-	+	-

## Discussion

In this study, we developed and evaluated real-time PCR and LAMP assays for the detection of DD associated treponemes. Current treponeme phylogroup PCR methods are conventional PCR and rely on size based band discrimination of the amplified PCR products [18,25]. Conventional PCR is more labor intensive than real-time PCR and LAMP assays due to the post PCR processing of gel electrophoresis and staining for visualization of the PCR products. Real-time PCR and LAMP assays are more precise, sensitive, and specific than conventional PCR methods [26,27]. Although nested PCR increases sensitivity it is also more labor intensive and is prone to contamination [28]. Therefore the development of treponeme phylogroup real-time PCR and LAMP assays was warranted.

The specificity of the developed real-time PCR and LAMP assays was evaluated with a test panel of 60 treponeme and non-treponeme control strains. The combination of the real-time primers and fluorescence-labeled probes and the LAMP primers designed in this study correctly detected all of the phylogroups with no cross-reactions and no amplification in any of the negative controls strains. The limit of detection for each of the three treponeme phylogroup real-time PCR assays and LAMP assays was ≤ 70 fg/μl. The detection limit for the *Treponema spp*. LAMP assay ranged from 7–690 fg/μl depending on phylogroup. The *Treponema spp*. LAMP assay’s efficiency is dependent on the phylogroup due to the primer design to encompass multiple treponeme species. The treponeme phylogroup real-time PCR assays and LAMP assays were equally specific and sensitive. The specificity of the PCR and LAMP assays is equivalent to the presently used treponeme phylogroup and *Treponeme spp*. PCR assays [18,25,29]. To our knowledge, there is no reported detection limits for the treponeme phylogroup PCR for comparison. However, detection limits for *Borrelia burgdorferri*, a spirochete relative, real-time PCR and LAMP assays were reported to be 50 fg/μl and 20–200 fg/μl, respectively [30,31]. These detection limits are similar to the detection limits of the developed treponeme PCR and LAMP assays in this study. These facts imply that the developed PCR and LAMP assays are useful tools for the detection of DD associated treponemes.

Real-time PCR assays have many advantages over conventional PCR including higher sensitivity and specificity, lower contamination rate and they are less time consuming. However, demanding expensive reagents and equipment restrict its application to some laboratories. LAMP assays are known as rapid, specific, sensitive, cost-effective and easy-operating alternative for the detection of pathogens. Therefore developing LAMP assays for detecting treponemes and differentiating the treponeme phylogroups is very useful. LAMP assays are capable of quantification but require additional detection instrumentation such as bioluminescence or fluorescence readers or a turbidimeter [32–34]. We chose to visualize the LAMP assays results with the naked eye for a qualitative interpretation keeping the assays practical and simple to use and cost effective. LAMP assays can be used in mobile laboratories, veterinary practices, and diagnostic laboratories [35]. The use of the *Treponeme spp*. LAMP assay for identifying treponemes in a sample and treponemes other than of the 3 specific phylogroups associated with DD is advantageous. In this study we isolated 2 treponeme isolates that were only positive for the *Treponeme spp*. LAMP assay, 16S rRNA sequencing revealed that both isolates closely aligned with *Treponema putidum*. This is further validation of the specificity of the developed phylogroup specific PCR and LAMP assays. Brandt et al. (2011) [29] used a real-time PCR targeting the 16S rRNA gene to recognize a broad panel of *Treponeme spp*. and then used conventional PCR and sequencing to identify treponeme phylogroups. This method is time-consuming, laborious and costly compared to using the developed *Treponeme spp*. LAMP assay to screen samples for treponemes followed by either the real-time PCR or LAMP phylogroup assays to identify the phylogroup.

Using a modified IMS method we were able to isolate treponemes from 40 DD lesion biopsies [21,22]. The PCR and LAMP assays were utilized for identifying treponeme phylogroups in the IMS cultures. The treponeme phylogroup real-time PCR and LAMP assay results had 100% agreement, matching on all samples (40/40). The 40 DD lesion IMS cultures in the current study were all positive for the *Treponeme spp*. LAMP assay and *T*. *pedis*, *T*. *phagedenis*, and *T*. *medium* phylogroup PCR/LAMP assays were positive for 35%, 85%, and 30% of the samples, respectively. Multiple phylogroups were detected in 48% of the IMS cultures. As already observed [10,12,36], this data further supports the association of multiple treponeme phylogroups with DD lesions in cattle. Although the modified IMS method used in this study was able to capture and isolate the three treponeme phylogroups associated with DD, certain features of the IMS method are worthwhile noting. Given the fastidious nature of treponemes, some of the IMS cultures remained mixed cultures even after numerous subculture attempts and 18/19 *T*. *pedis* and *T*. *medium* cultures were unable to be grown back onto solid media. Treponemes are known to adhere to themselves, other bacteria, and microparticles as a survival mechanism [37–39]. This could explain the mixed cultures in this study, the treponeme and bacteria complexes are being captured together by the IMS method and lead to mixed cultures. Evans et al. (2008) [25] suggest using phylogroup specific growth conditions and supplements to obtain pure treponeme isolates and to solve this problem.

The primary focus of this study was to isolate treponemes from DD lesion biopsies therefore the IMS/culture method was used to enhance isolation. The IMS/culture method confirms that the treponemes are viable since the real-time PCR and LAMP assays cannot distinguish between viable and nonviable treponeme DNA. In a pilot phase of this study the real-time PCR and LAMP assays detected treponemes from DNA extracted directly from the biopsy transport media, enriched biopsy media and lesion biopsies (data not shown). Therefore, the developed real-time PCR and LAMP assays are capable of detecting and quantifying treponemes extracted directly from the DD lesion biopsies without limitations for future studies.

In conclusion the real-time PCR and LAMP assays developed in this study can enhance a researcher’s ability to detect and identify the three treponeme phylogroups associated with DD from any sample with equal diagnostic accuracy. The only notable difference between the real-time PCR and LAMP assays is that the LAMP assays are more cost effective due to the LAMP assays only requiring a low cost heat block to perform. These real-time PCR and LAMP assays may facilitate detection and quantification of the treponeme phylogroups in relation to DD transmission and can be applied to study the pathogenesis of DD.

## Supporting information

S1 Fig Real-time PCR treponeme phylotype standard curves. (PDF)

S1 Table Specificity results for the real-time PCR and LAMP assays. (PDF)
