# Supplementary material for: Development of real-time PCR and loop-mediated isothermal amplification (LAMP) assays for the differential detection of digital dermatitis associated treponemes
Source: PLoS One. 2017 May 25;12(5):e0178349. doi: 10.1371/journal.pone.0178349 (PMC5444799; doi:10.1371/journal.pone.0178349)
Supplement: S1 Fig — (PDF) [file pone.0178349.s001.pdf]

S1 Figure. Real-time PCR treponeme phylotype standard curves.

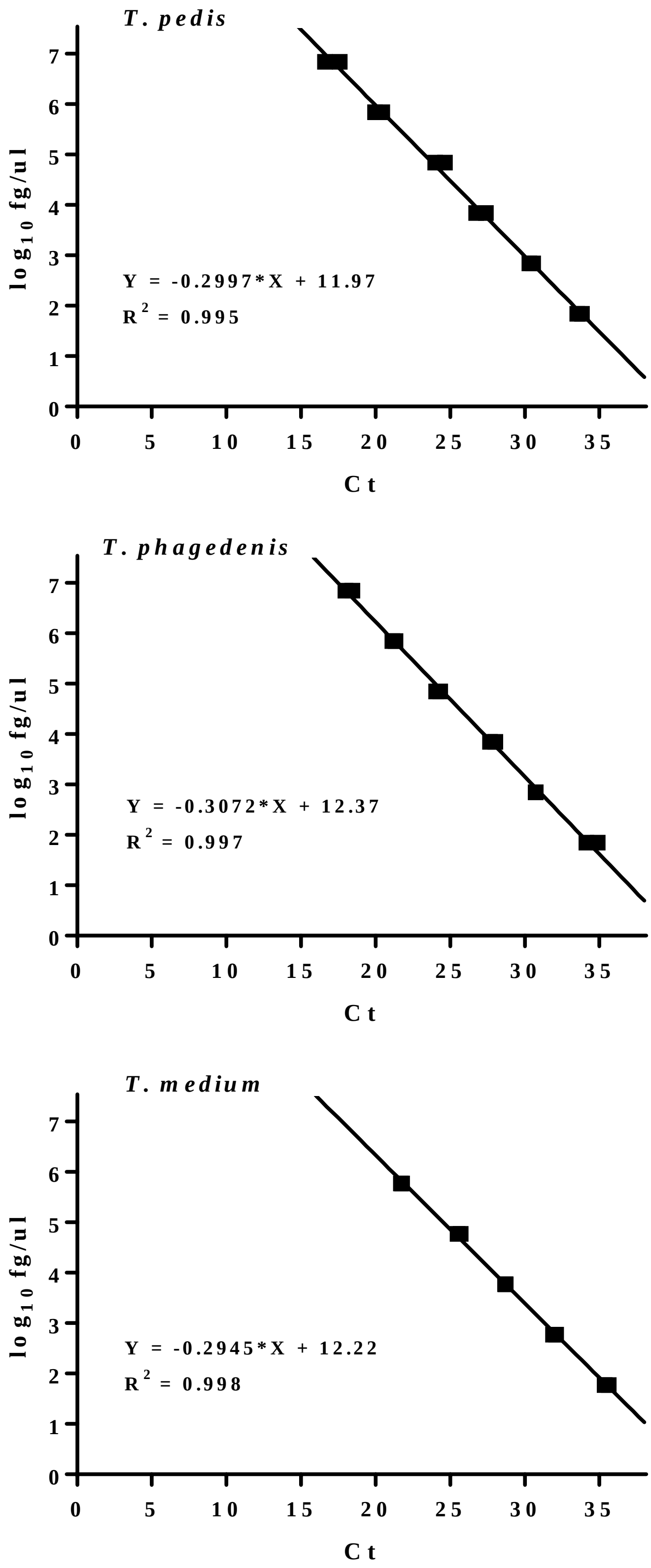

S1 Figure. Standard curves were determined by plotting the Ct value obtained from amplification of each phylotype target sequence against DNA concentration (log fg/µl) for each dilution. Ct represents the real-time PCR cycle at which fluorescence reaches the threshold value.
