# Supplementary material for: Development of real-time PCR and loop-mediated isothermal amplification (LAMP) assays for the differential detection of digital dermatitis associated treponemes
Source: PLoS One. 2017 May 25;12(5):e0178349. doi: 10.1371/journal.pone.0178349 (PMC5444799; doi:10.1371/journal.pone.0178349)
Supplement: S1 Table — (PDF) [file pone.0178349.s002.pdf]

S1Table. Specificity results for the real-time PCR and LAMP assays.

#	Bacterial Strain	***T. species* LAMP**	***T. pedis* LAMP**	***T. phagedenis* LAMP**	***T. medium* LAMP**	***T. pedis* PCR**	***T. phagedenis* PCR**	***T. medium* PCR**
1	*T. pedis* 1-9185	+	+	-	-	+	-	-
2	*T. pedis* AP	+	+	-	-	+	-	-
3	*T. pedis*AW	+	+	-	-	+	-	-
4	*T. pedis* tA	+	+	-	-	+	-	-
5	*T. pedis* tB	+	+	-	-	+	-	-
6	*T. pedis* CA	+	+	-	-	+	-	-
7	*T. phagedenis* 2-1498	+	-	+	-	-	+	-
8	*T. phagedenis* AF	+	-	+	-	-	+	-
9	*T. phagedenis* AH	+	-	+	-	-	+	-
10	*T. phagedenis* AT	+	-	+	-	-	+	-
11	*T. phagedenis* AV	+	-	+	-	-	+	-
12	*T. phagedenis* BA	+	-	+	-	-	+	-
13	*T. phagedenis* AC	+	-	+	-	-	+	-
14	*T. phagedenis* AM	+	-	+	-	-	+	-
15	*T. phagedenis* CD	+	-	+	-	-	+	-
16	*T. phagedenis* CV	+	-	+	-	-	+	-
17	*T. phagedenis* CG	+	-	+	-	-	+	-
18	*T. phagedenis* CZ	+	-	+	-	-	+	-
19	*T. phagedenis* IA	+	-	+	-	-	+	-
20	*T. phagedenis* 977	+	-	+	-	-	+	-
21	*T. phagedenis* 221	+	-	+	-	-	+	-
22	*T. phagedenis* 749	+	-	+	-	-	+	-
23	*T. phagedenis* T28	+	-	+	-	-	+	-
24	*T. phagedenis* 224	+	-	+	-	-	+	-
25	*T. medium* 923L	+	-	-	+	-	-	+
26	*T. medium* CL	+	-	-	+	-	-	+
27	*T. medium* CN	+	-	-	+	-	-	+
28	*T. medium* CO	+	-	-	+	-	-	+
29	*T. medium* DB	+	-	-	+	-	-	+
30	*T. medium* 916M	+	-	-	+	-	-	+
31	*T. medium* 916N	+	-	-	+	-	-	+
32	*T. medium* 916T	+	-	-	+	-	-	+
33	*T. medium* 923D	+	-	-	+	-	-	+
34	*T. medium* 923K	+	-	-	+	-	-	+
35	*Bacillus cereus*	-	-	-	-	-	-	-
36	*Bacillus fragilis*	-	-	-	-	-	-	-
37	*Borrelia burgdorferi*	-	-	-	-	-	-	-
38	*Brachspira hyodysenteriae*	-	-	-	-	-	-	-
39	*Dichelobacter nodosus*	-	-	-	-	-	-	-
40	*Fusobacterium necrophorum*	-	-	-	-	-	-	-
41	*Fusobacterium nucleatum*	-	-	-	-	-	-	-
42	*Porphyromonas levii*	-	-	-	-	-	-	-
43	*Streptococcus dysgalactiae*	-	-	-	-	-	-	-
44	*Prevotella denticola*	-	-	-	-	-	-	-
45	*Citrobacter freundii*	-	-	-	-	-	-	-
46	*Enterobacter taylorae*	-	-	-	-	-	-	-
47	*Entrobacter aerogenes*	-	-	-	-	-	-	-

48	*Enterococcus feacalis*	-	-	-	-	-	-	-
49	*Escherichia coli* K-12	-	-	-	-	-	-	-
50	*Escherichia coli* O157	-	-	-	-	-	-	-
51	*Klebsiella ozanae*	-	-	-	-	-	-	-
52	*Leptospira interrogans* B	-	-	-	-	-	-	-
53	*Leptospira interrogans* G	-	-	-	-	-	-	-
54	*Leptospira interrogans* H	-	-	-	-	-	-	-
55	*Listeria moncytogenes*	-	-	-	-	-	-	-
56	*Pseudomonas aeruginosa*	-	-	-	-	-	-	-
57	*Salmonella enteriditis*	-	-	-	-	-	-	-
58	*Staphylococcus aureus*	-	-	-	-	-	-	-
59	*Shigella dysenteriae*	-	-	-	-	-	-	-
60	*Yersinia enterocolitica*	-	-	-	-	-	-	-
